# Effects of Microstructured and Anti-Inflammatory-Coated Cochlear Implant Electrodes on Fibrous Tissue Growth and Neuronal Survival

**DOI:** 10.3390/jfb16010033

**Published:** 2025-01-18

**Authors:** Lennart Fibranz, Wiebke Behrends, Katharina Wulf, Stefan Raggl, Lisa Kötter, Thomas Eickner, Soeren Schilp, Thomas Lenarz, Gerrit Paasche

**Affiliations:** 1 Department of Otorhinolaryngology, Hannover Medical School, 30625 Hannover, Germany; lennart.fibranz@stud.mh-hannover.de (L.F.); koetter.lisa@mh-hannover.de (L.K.); lenarz.thomas@mh-hannover.de (T.L.); 2 Lower Saxony Center for Biomedical Engineering, Implant Research and Development (NIFE), Hannover Medical School, 30625 Hannover, Germany; 3 Institute for Biomedical Engineering, Rostock University Medical Center, 18119 Rostock, Germany; katharina.wulf@uni-rostock.de (K.W.); thomas.eickner@uni-rostock.de (T.E.); 4 MED-EL Medical Electronics, 6020 Innsbruck, Austria; stefan.raggl@medel.com (S.R.); soeren.schilp@medel.com (S.S.); 5 Hearing4all Cluster of Excellence, Hannover Medical School, 30625 Hannover, Germany

**Keywords:** surface patterns, microstructures, diclofenac, connective tissue, polymeric coating, spiral ganglion cell survival

## Abstract

Cochlear implants are well established devices for treating severe hearing loss. However, due to the trauma caused by the insertion of the electrode and the subsequent formation of connective tissue, their clinical effectiveness varies. The aim of the current study was to achieve a long-term reduction in connective tissue growth and impedance by combining surface patterns on the electrode array with a poly-L-lactide coating containing 20% diclofenac. Three groups of six guinea pigs each (control, structure, structure with diclofenac in the coating) were implanted for four weeks. The hearing thresholds were measured before implantation and after 28 days, and impedances were monitored over time. After histological preparation, connective tissue growth and spiral ganglion neuron (SGN) survival were quantified. The hearing thresholds and impedances increased over time in all groups, showing no significant differences. The treatment groups showed increased damage in the cochlea, which appeared to be caused by the elevated parts of the microstructures. This seems to be amplified by the trauma model used in the current study. The impedances correlated with connective tissue growth near the electrode contacts. In addition, SGN survival was negatively correlated with the presence of connective tissue, both of which highlight the importance of successfully reducing connective tissue formation after cochlear implantation.

## 1. Introduction

Treatment of severe to profound hearing loss with cochlear implants (CI) is well established. The CI electrode transmits incoming information to the auditory cortex by electrically stimulating the spiral ganglion neurons (SGNs). However, insertion of the electrode usually causes mechanical trauma, damaging intracochlear tissue and resulting in an ongoing inflammatory response. Consequently, residual acoustic hearing can be impaired. These effects show that, despite the benefits of cochlear implantation, the clinical effectiveness varies widely between patients.

The preservation of residual acoustic hearing and CI success may be increased by enhancing the SGN survival. Studies have shown that hearing and word recognition in CI patients are dependent on the number of SGNs in the cochlea [1,2]. However, the survival of SGNs is highly dependent on electrical stimulation. Therefore, it is important that the nerve–electrode interface is as unaffected as possible [3]. As connective tissue is known to increase electrode impedances when it surrounds the electrode contacts, the reduction of post-implantation connective tissue growth supports hearing preservation by allowing better stimulation of the SGNs [4]. This is consistent with the observation that compact tissue worsens the interface between the electrode and the neurons, resulting in poorer stimulation of the SGNs [5].

The insertion of the electrode causes trauma that triggers an inflammatory reaction, resulting in the formation of connective tissue varying in size from a few cells to compact new bone formation [6]. Additionally, the connective tissue, as well as the SGN density, depend on the trauma caused [7]. Therefore, minimizing trauma during electrode insertion has been investigated in many different experimental approaches. Studies showed that the insertion trauma depends on the insertion technique, and that insertion through the round window is less traumatic than a cochleostomy [8,9]. Additionally, smaller and more flexible electrodes tend to cause less trauma to the cochlea and therefore, reduce the damage during implantation [10].

However, the fact that the insertion is a traumatic event for the cochlea does not change. Therefore, many studies using different anti-inflammatory substances have been conducted to inhibit the inflammatory response after implantation. In particular, glucocorticoids have been investigated for use in the cochlea. Studies showed inhibitory effects on inflammatory reactions after cochlear implantation in vivo, as well as reduced impedances up to 2 years after surgery in a clinical trial when using glucocorticoids [11,12]. The glucocorticoid dexamethasone (DEX) is considered safe for inner ear treatment, as ototoxic effects were ruled out [13]. Therefore, the treatment with DEX is well established in CI treatment. Current clinical studies also analyzed how DEX incorporated into the silicone body of the electrodes helped preserve hearing. The results showed lower impedances, confirming the high potential of DEX in CI treatment [14]. Next to glucocorticoids, diclofenac also showed promising results in the inhibition of fibrous tissue as well [15,16]. In vitro experiments determined that diclofenac has no toxic effects on SGNs up to a concentration of 4 × 10^−5^ mol/L [17]. Diclofenac was used once in vivo in the cochlea and did not appear to have any negative effects on the SGNs [18].

In addition, many drug-application methods have been suggested and are under assessment. Local and systemic DEX application, pumps, hydrogels on the round window, and drug-loaded coatings were analyzed to find the best way to treat the inner ear using anti-inflammatory drugs [19,20,21,22,23]. Loadable and biodegradable polymeric coatings have proven to be promising drug reservoirs. In vitro studies showed that biodegradable coatings release the incorporated drug faster and reach higher concentrations than do DEX-loaded silicone arrays [24]. The use of local dual-drug delivery systems (LDD) ensures both an initial high dose release and a constant, long-term release of anti-inflammatory substances. Poly-L-lactide (PLLA) seems to be a promising coating polymer for dual-drug delivery [17], as it neither negatively affects SGN survival nor leads to higher implantation forces in a linear scala tympani model compared to the use of pure silicone arrays [25,26]. As PLLA is already used in cardiovascular stents, we decided to use it in the current study [27].

Surface structures influence the attachment and orientation of fibroblasts [28]. Cell morphology, and proliferation rate can be altered by microstructured polymers [29]. With the use of structured silicone, fibroblasts tended to round up and detach from the surface. This was dependent upon the size of the structures and was associated with decreased fibroblast growth [30]. In addition, surface modifications on platinum electrodes resulted in lower impedances for a short time after implantation in vivo [31]. Therefore, surface modifications on CI electrodes may be able to reduce fibrosis; however, no long-term effect has yet been proven.

In the current study, due to the missing long-term effect of surface patterning alone, this approach was combined with a PLLA-coating of the electrode array and additional incorporation of DEX in the silicone body of the electrode; the results were then compared to those for DEX-loaded electrodes, with and without surface patterning. The study was carried out in guinea pigs, as these animals have been used as the standard model for the preclinical implantation of multichannel CI electrodes for decades (compare with [32]).

## 2. Materials and Methods

### 2.1. Ethical Statement and Experimental Design

The study was acknowledged and approved by the State Office for Consumer Protection and Food Safety, Dept. of Animal Welfare, under the number 20/3502. The animals were kept in the Lower Saxony Center for Biomedical Engineering, Implant Research, and Development (NIFE) under a 14/10 h light/dark cycle. They had free access to food and drinking water at all times. The experiments were conducted in compliance with the legal directives for accommodation, care, and usage of experimental animals.

The unilateral implantation of CI was performed on 18 male Dunkin Hartley guinea pigs (Charles River Laboratories, Châtillon, France). The animals were divided into three equally sized groups, receiving differently modified CI electrode arrays. The duration of the study was 28 days and started the day of CI implantation, after confirmation of normal hearing. Measurements of impedances were taken daily for the first 14 days and then weekly for two additional weeks. On day 28, the animals were anesthetized, and hearing thresholds were determined before the final measurements were taken. While still under anesthesia, the animals were euthanized using transcardial perfusion with 200 mL of phosphate-buffered saline (PBS, Gibco™, Thermo Fisher Scientific, Paisley, UK), followed by 100 mL of 4% paraformaldehyde (PFA, Merck KGAA, Darmstadt, Germany). Finally, the animals were decapitated, and both cochleae were dissected from the temporal bones. The CI electrodes remained inside the left cochlea. After histological preparation, the cochleae were scanned and analyzed, along with the rest of the data.

### 2.2. Electrode Arrays

**Figure 1:**
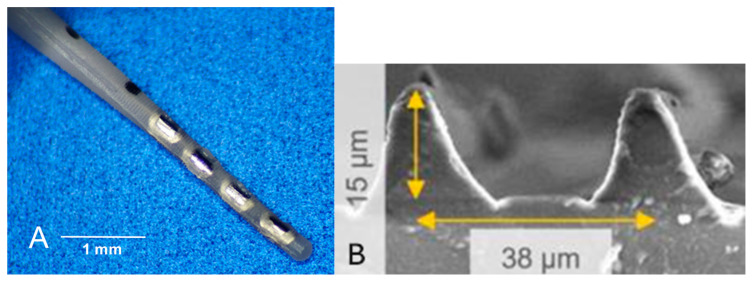
(**A**) Electrode array with platinum contacts and the two markers at 3 and 4 mm from the tip. (**B**) Raster electron microscopic image of the microstructures.

Animal-sized CI electrodes (provided by MED-EL GmbH, Innsbruck, Austria), with four platinum contacts at the distal tip of the electrode array and an additional connector, as well as a reference electrode, were used for implantation. Two black marker dots were added at 3 mm and 4 mm from the tip of the array to control the depth of insertion (Figure 1A). All electrode arrays were made of silicone containing 5% DEX (Sanofi, Paris, France). The first group, called “Control”, received the otherwise untreated electrode arrays. Electrodes of the first treatment group were characterized by a circular microstructure on the silicone array. This group is referred to as “Struc”. The circular grooves (38 µm width, 15 µm depth, see Figure 1B) for the treatment groups were created by using specially microstructured molds and a subsequent silicone injection process. In addition to the circular microstructure, the electrodes of the third group (“StrucDic”) were coated with PLLA, which was loaded with diclofenac (PLLA/diclofenac 80:20 wt%). To prevent the contacts from being coated, masks were attached to the surface prior to the PLLA coating process. Afterwards, the masks were removed, as described by Wulf et al. [17].

### 2.3. Coating Process

The StrucDic electrode arrays were coated following the protocol established by Wulf et al. and Behrends et al. 2023 [17,18]. First, the silicone surface of the electrode was activated by means of O_2_ plasma at 100 W and 0.3 mbar pressure for 1 min in a plasma chamber (Diener, Ebhausen, Germany). After that, the silicone was incubated in pure GOPS (3-glycidyloxypropyl-trimethoxysilane, Merck KGAA) for 6 h at 90 °C. The incubated and activated samples were rinsed three times in pure ethanol and then dried at 80 °C overnight in a 40 mbar vacuum. Afterwards, in an established and in-house manufactured spray coating process, the activated electrodes were spray-coated with a thin layer of PLLA-NH2 (VWR, Dresden, Germany). For this step, a chloroform PLLA-NH2 (2 wt%) spray solution was used. Once again, the samples were dried overnight at 80 °C in a 40 mbar vacuum. The electrodes were then coated with a chloroform PLLA (2 wt%) spray solution containing diclofenac sodium salt (Merck KGAA) at a ratio to PLLA of 20:80 wt%. A layer thickness of about 10 µm, corresponding to 70 µg coating mass per electrode array, was achieved. At the end, the contact masks, protecting the contacts during the coating process, were removed.

### 2.4. Implantation

For implantation surgery, the animals were put under general anesthesia (intramuscular medetomidine hydrochloride 0.2 mg/kg (alfavet Tierarzneimittel GmbH, Neumuenster, Germany), midazolam 1 mg/kg (Panpharma GmbH, Trittau, Germany), and fentanyl 0.025 mg/kg (Dechra Veterinary Products Deutschland GmbH, Aulendorf, Germany), in combination with a previous sedation with oral diazepam 4 mg/kg (ratiopharm GmbH, Ulm, Germany)). Cochlear implantation was performed on the left side for all guinea pigs. The surgery started by locally infiltrating the surgical field with prilocaine (Xylonest 1%, Aspen Germany GmbH, Munich, Germany). The skin was incised, and the top of the skull was dissected. The CI electrode, as well as the reference electrode, were directed underneath the skin and muscles towards the bulla tympanica. The bulla was opened using an approach from behind the ear [18]. The cochlea then laid exposed in the bulla. A 0.7 mm diameter cochleostomy was drilled (AccuPen 6V+; RISystem AG, Landquart, Switzerland) into the cochlea 1 mm below the round window. Insertion of the CI electrode into the cochlea was performed by using an insertion trauma approach previously described by Wilk et al. [4]. All electrodes were inserted up to the second marker dot on the electrode array, confirming a 4 mm insertion of the active electrode array. After that, the electrodes were fixed with UV dental cement (Tetric EvoFlow^®^, Ivoclar Vivadent, Ellwangen, Germany) to the edge of the bulla tympanica. Screws and methyl methacrylate (Paladur^®^, Kulzer GmbH, Hanau, Germany) were used to attach the connector to the skull. Afterwards, the muscle layers and the skin layers were sutured. The implantation surgery was completed by antagonizing the anesthesia with naloxone (0.03 mg/kg s.c., naloxone, Inresa Arzneimittel GmbH, Freiburg, Germany), flumazenil (0.1 mg/kg s.c., flumazenil, Hameln pharma GmbH, Hameln, Germany), and atipamezole (1.0 mg/kg s.c., atipazole, Prodivet pharmaceuticals sa/nv, Eynatten, Belgium). The animals were positioned under a heating lamp until they were fully recovered from anesthesia.

### 2.5. Auditory Brainstem Response Measurements

Auditory brainstem response (ABR) measurements were performed under general anesthesia in a sound attenuating chamber using a TDT system (Tucker-Davis Technologies, Alachua, FL, USA) on day 0 before implantation and on day 28 before sacrifice. An EC1 Speaker (Tucker-Davies-Technology) was placed in the outer ear canal, creating acoustic stimulations. The frequencies 1 kHz, 2 kHz, 4 kHz, 8 kHz, 16 kHz, 32 kHz, and 40 kHz were stimulated at sound pressure levels from 100 dB down to 0 dB in 5 dB steps. Acoustic tone stimuli with 10 pulses per second, a duration of 10 ms, and a square cosine rise and fall time of 1 ms were used. The neurological answers were derived and recorded by subdermal needle electrodes (CareFusion Nicolet, Middleton, WI, USA) at the vertex (common positive), left and right mastoid (references), and the neck (ground). Before analysis, the neurological signals were sampled 300 times and averaged using the BioSigRP-software(Version 4.4.1) prepared for the TDT system. Additionally, the recorded signals were bandpass filtered from 300 to 3000 Hz to suppress background noises. The hearing threshold was defined as the lowest sound stimulus required to evoke a visually replicable waveform. If no threshold was detected, 100 dB was used for analysis.

### 2.6. Impedance Measurements

The electrode impedance measurements were performed in monopolar mode using the impedance field telemetry (IFT) task of the MED-EL clinical system, consisting of a MAX-Box and the MAESTRO software (Version 8.0). Impedance data was collected for each contact daily for the first 14 days and weekly thereafter (day 21 = week 3; day 28 = week 4). Measurements higher than the device’s limit (open circuits) were displayed with the value of 21 kOhm. The data were analyzed separately for each contact and each day. Invalid data, such as due to open circuits or incorrectly positioned electrodes, were discarded from the analysis.

### 2.7. Histology

Histological preparation followed a modified protocol from MacDonald and Rubel [33]. Firstly, the dissected cochleae were fixed overnight in PFA 4%. After that, the cochleae were rinsed three consecutive times in PBS for 10 min. For decalcification, the cochleae were stored in a solution of 10% ethylenediaminetetraacetic acid disodium salt (EDTA; Sigma-Aldrich Chemie GmbH, Schnelldorf, Germany) in PBS (pH 7.4) for three to four weeks. The decalcified cochleae were then rinsed in PBS for ten minutes for three consecutive times. In the next step, the cochleae were stored in a solution with Triton X-100 (Sigma-Aldrich Chemie GmbH) 1:100 dissolved in PBS for six to eight hours at room temperature. In preparation for staining, the cochleae were incubated for three days at 4 °C with the primary antibody (Anti-Vimentin antibody produced in goat, Sigma-Aldrich Chemie GmbH), which was diluted in blocking solution containing 5% normal horse serum (Biozol, Eching, Germany) and 1% Triton X-100 in PBS. To stop staining, the samples were washed with PBS three times for two hours each. After that, the cochleae were incubated for three days at 4 °C in Alexa Fluor 647-conjugated (AffiniPure Bovine Anti-Goat IgG, Jackson ImmunoResearch, West Grove, PA, USA) as a secondary antibody. Following another washing cycle in PBS, dehydration was performed in ethanol of an ascending concentration on a platform rotator at the following rates: 75% (overnight), 90% (for 30 min), and 100% (2 h). Temporary incubation of the cochleae for 4 h in a 1:1 mix of MSBB (methyl salicylate benzyl benzoate, Merck KGAA) and 100% ethanol on a platform rotator ensured a tissue-friendly transition of the cochleae to permanent storage. The permanent storage and adjustment of the refractive index was carried out in pure MSBB at a temperature of 4 °C.

### 2.8. Imaging

A Leica SP8 laser scanning confocal microscope (Leica Microsystems GmbH, Wetzlar, Germany) was used to scan the cochleae. The microscope was equipped with a white light laser and an objective lens with 10× magnification (HC PL Fluotar 10×/0.30 Dry, Leica Microsystems GmbH). The software (LAS X Science Microscope Software, version LAS X 3.5.7.23225) allowed for the control of the laser settings and the visualization of the scans. Excitation lines with wavelengths at 492 nm (for PFA-induced autofluorescence, channel 1) and 652 nm (for Alexa Fluor 647, channel 2) were used. Scans were performed with slices of 20 µm (z-stack on) at a scanning speed of 400 Hz, with 5× line averaging and 3× frame averaging. To be able to see the electrode, the TLD (bright field detector/through the lens detector) was used, and it produced a third channel.

### 2.9. Connective Tissue Quantification

Two custom-made subjective ranking scores [18] were used to evaluate the tissue growth in the cochlea. Seven different sections of the cochlea were examined: lower basal turn (lb), upper basal turn (ub), first middle turn (1st), second middle turn (2nd), third middle turn (3rd), fourth middle turn (4th), and apical turn (ap). The first score rates the newly formed tissue in all cochlea turns, depending on the percentage of the respective cross-sectional scala tympani area filled with connective tissue. A score of 0 indicates no connective tissue at all, whereas a score of 4 indicates a turn completely filled with connective tissue. When up to 25% of the turn is filled with connective tissue, a score of 1 is given (up to 50% = a score of 2; more than 50% = a score of 3).

**Figure 2:**
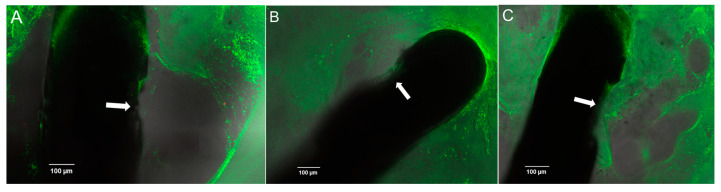
Examples of contact-score ratings. (**A**) Score 1 (StrucDic3); (**B**) Score 2 (StrucDic8); (**C**) Score 3 (Control9). Small arrows point to the contacts.

The second score assesses the connective tissue directly around the contacts of the CI electrode (contact-score): 0 is defined as no connective tissue, 1 = a thin film of tissue directly on the contact (Figure 2A), 2 = reticular tissue at the contact (Figure 2B), 3 = the contact completely covered by compact connective tissue (Figure 2C).

### 2.10. Spiral Ganglion Neuron Counting

SGNs were automatically counted using the ITCN plug-in (Image-based Tool for Counting Nuclei, Center for Bio-Image Informatics; https://bioimage.ucsb.edu/docs/automatic-nuclei-counter-plugin-imagej, installed on 1 August 2022) for ImageJ software (Wayne Rasband, National Institutes of Health, Bethesda, MD, USA). In contrast to the quantification of the connective tissue, the 4th middle and apical turns were counted as one section. Five mid-modiolar regions were selected for each of the six sections of the cochlea. First, in accordance with the protocol described by Wrzeszcz, the perimeters of Rosenthal’s canal were outlined by hand [34]. In the marked area, neurons with a diameter larger than 17 µm were counted. Additionally, a minimum distance of 8.5 pixels between two cells, as well as a threshold of 0.1, were defined in the settings [34]. SGN density, expressed as cells/10,000 µm^2^, was determined by relating the number of vital SGNs to the marked cross-sectional area of Rosenthal’s canal. For the slices in which Rosenthal’s canal was covered by shadows cast by the electrode, no SGN count was performed. This affected slices of four animals in the StrucDic group, three animals in the Struc group, and none in the Control group.

### 2.11. Different Plug-In Versions

The ITCN plug-in for the ImageJ software can be downloaded from the website of the Center for Bio-Image Informatics (UC Santa Barbara, Santa Barbara, CA, USA), as well as from the official ImageJ website (Wayne Rasband, National Institutes of Health, Bethesda, MD, USA). Both plug-ins enable the automated counting of the number of SGNs. The plug-in provided by the Center for Bio-Image Informatics offers the opportunity of defining a threshold of 0.1. This setting was not found in the other plug-in. Due to this difference, the results of both settings were compared to an additional manual count using the cochleae of the Control group. The results are presented as absolute values, as the same cross-sectional area was examined.

### 2.12. Data Evaluation and Statistics

GraphPad Prism 10 (GraphPad Software; Boston, MA, USA) was used to perform the data evaluations. First, data were tested for normal distribution using the Shapiro–Wilk test. Hearing thresholds were analyzed using either unpaired *t*-tests or Mann–Whitney tests, depending on the results of the normality test. One-way ANOVA or mixed effects analysis was performed for impedance measurement analysis, depending on the completeness of the data sets. Kruskal–Wallis tests were performed to compare the three groups regarding their newly formed connective tissue in each turn. Subsequently, the mean values of the turns’ connective tissue scores were compared between the groups using unpaired *t*-test and Wilcoxon tests. For SGN density analysis, the control side was first compared to the implanted left side using paired *t*-tests or Wilcoxon matched-pairs tests. Then, the groups were compared to each other using unpaired *t*-tests or Mann–Whitney tests. To analyze the results with the two different ITCN plug-ins, data obtained from the manual count, the automatic count with the 0.0 threshold, and the automatic count with the 0.1 threshold were analyzed using the Friedman test. Potential correlations between SGN density and the connective tissue score were analyzed using linear regression. The data from all 18 animals and their turns were considered. Linear regression was used to analyze potential correlations between the impedance measurements and the corresponding electrode contact connective tissue scores. Only complete data sets, including both impedance values and associated contact scores, were considered. This resulted in *n* = 15 for contacts 1 and 2 and *n* = 11 for contact 3. Significant differences were indicated by *p*-values below 0.05.

## 3. Results

### 3.1. Comparison Between the Two Plug-Ins

**Figure 3:**
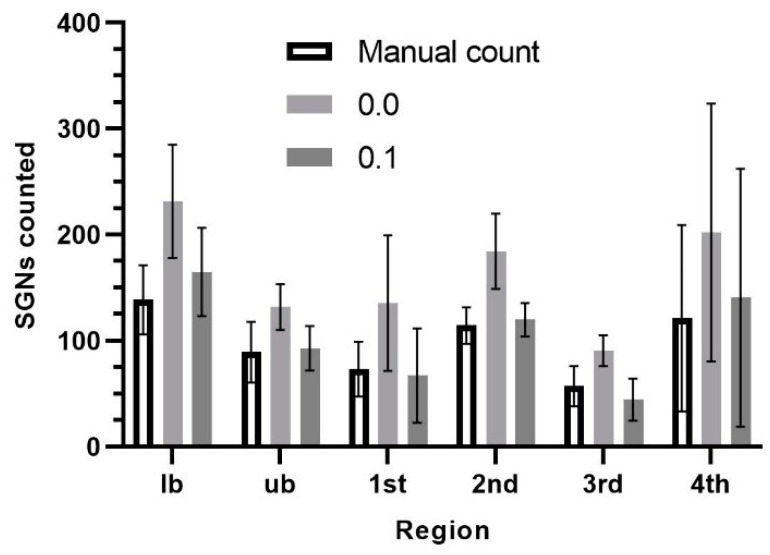
Number of spiral ganglion neurons (SGNs) for different cochlear regions and the different counting methods.

For comparison of the three different counting options, mean values of the counted SGNs in the six cochleae were analyzed for each region. In the manual count, 99 ± 49 cells (mean ± SD) per section were counted, on average, over all six regions and all animals. The cell count with the 0.1 threshold resulted in an average of 105 ± 68 cells. Using the plug-in without the threshold setting, the mean SGN count was 163 ± 76 cells. The latter value was significantly higher than both other values (*p* < 0.0001 for threshold 0.1 and *p* = 0.001 for the manual count). No difference between the 0.1 threshold and the manual count was detected (*p* = 0.3742). Accordingly, the highest cell counts in each section were detected when using the 0.0 threshold. The lowest cell count per region was found in the 1st middle turn and the 3rd middle turn using the 0.1 threshold. In the other four regions, the manual count resulted in the lowest cell counts (Figure 3). Analysis of the different thresholds resulted in a factor of 0.63 for the entire cochlea to convert the values from the 0.0 setting to the 0.1 setting. The factors differed between the individual turns. The basal turns showed very similar factors of 0.71 (lb) and 0.72 (ub). In the first middle turn and the 3rd middle turn, the factor was 0.5. The second middle turn showed a factor of 0.65, and the data of the fourth middle turn, combined with the apical turn, resulted in a factor of 0.7.

### 3.2. Acoustically Evoked ABR Hearing Thresholds

**Figure 4:**
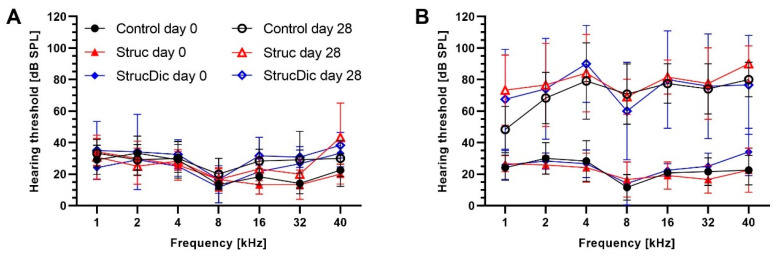
Hearing threshold on the unimplanted Control (**A**) and on the implanted side (**B**) on days 0 and 28.

**Figure 5:**
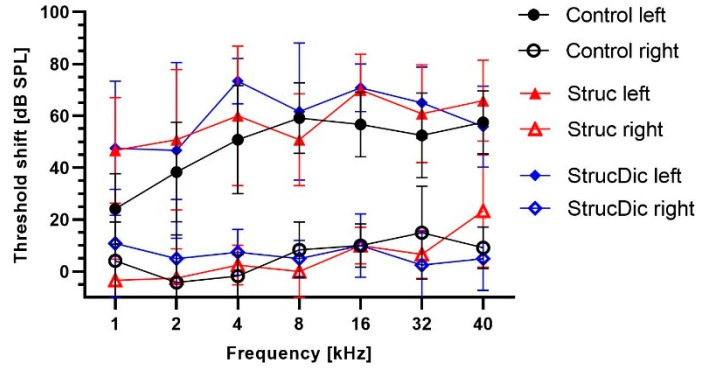
Threshold shifts at different frequencies on implanted (filled symbols) vs. unimplanted (open symbols) sides.

Before implantation, all guinea pigs exhibited normal hearing (threshold below 50 dB), with no differences between sides. Until day 28, the hearing threshold increase was small in all three groups on the control side (Figure 4A). On the implanted side, the hearing thresholds increased strongly until day 28 (Figure 4B). The average threshold shifts on the implanted side were 49 dB ± 11 dB (Control), 57 dB ± 11 dB (Struc), and 59 dB ± 13 dB (StrucDic) on day 28. In contrast, on the unimplanted side, the average hearing thresholds were elevated by 6 dB ± 7 dB (Control), 5 dB ± 9 dB (Struc), and 7 dB ± 3 dB (StrucDic) on day 28 (Figure 5). The differences between the implanted and unimplanted sides were significant (*p*-values between 0.0001 for Control 16 kHz and 0.0285 for Struc 40 kHz) in all groups and frequencies. The only exceptions were found at 1 kHz in the Control group (*p* = 0.515) and the StrucDic group (*p* = 0.0536) and at 2 kHz in animals of the StrucDic group (*p* = 0.0768).

When comparing the hearing loss between the three groups for each frequency on the implanted side, significant differences were only found between the Control group and StrucDic at 4 kHz (*p* = 0.0349) and at 16 kHz (*p* = 0.0493). At both frequencies, StrucDic showed a larger hearing loss (4 kHz = 73 dB; 16 kHz = 71 dB) than did the Control group (4 kHz = 51 dB; 16 kHz = 57 dB).

### 3.3. Electrode Impedances

**Figure 6:**
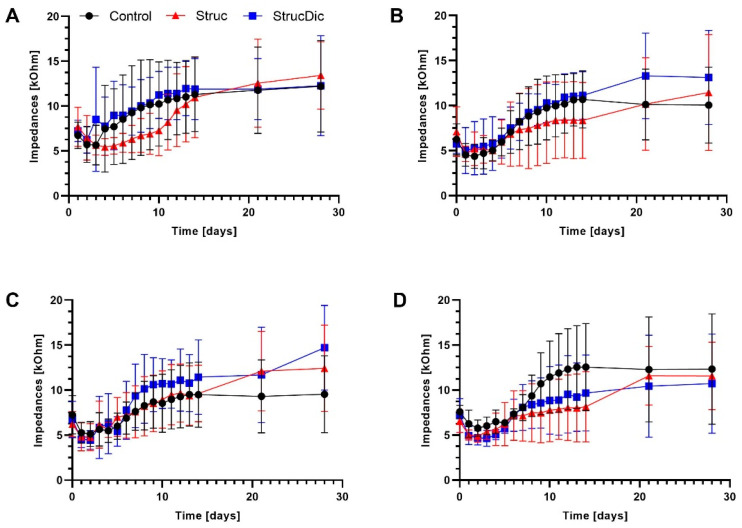
Electrode impedance measurements from day 0 to day 28. (**A**) contact 1, (**B**) contact 2, (**C**) contact 3, (**D**) contact 4. Mean ± standard deviation (SD). Contact 4 (**D**) is closest to the cochleostomy, contact 1 (**A**) is the most apical contact.

On the day of implantation, the average impedances of all contacts were higher (Control: 6.92 ± 1.57 kOhm; Struc: 6.91 ± 1.97 kOhm; StrucDic: 6.72 ± 1.58 kOhm) than on the following days (Figure 6A–D). The lowest impedances were measured on day 1 in the two treatment groups (Struc: 5.24 ± 1.66 kOhm; StrucDic: 5.22 ± 1.67 kOhm) and on day 2 in the Control group (5.25 ± 1.73 kOhm). The impedances increased over time up to 10.98 ± 4.75 kOhm (Control), 12.26 ± 4.54 kOhm (Struc), and 12.53 ± 5.13 kOhm (StrucDic) across all contacts on day 28 (Figure 6A–D). This increase over time is significant in all groups for all four contacts. There was no significant difference between the groups at day 28 (*p* = 0.5044, mixed-effects analysis). Even though Figure 6A indicates a delayed increase in impedances at contact 1 in the Struc group, there were no significant differences between the three groups on any day.

### 3.4. Connective Tissue Quantification

**Figure 7:**
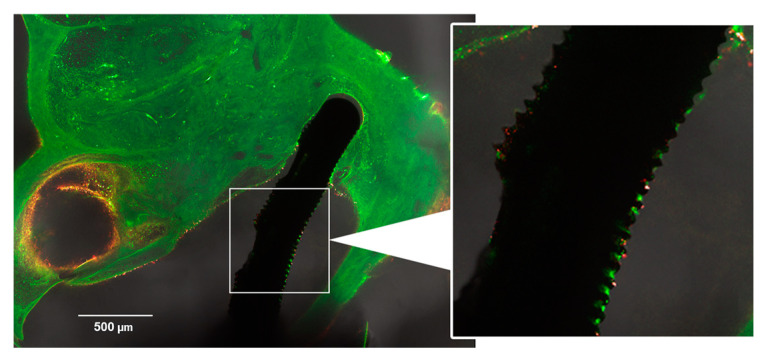
Example for extended damage (electrode tip in scala vestibuli after disrupting basilar membrane). Notify the visible microstructures as well as the little connective tissue in vicinity of the electrode as long as it is located in scala tympani (animal: Struc6).

Although all implantations were consistently performed by the same person using the same procedure, there were differences between the groups regarding the position of the electrode. In some cases, the electrode was found in the scala vestibuli or even damaged the second turn (i.e., extended damage; compare Figure 7). This extended damage was found with one of the electrodes in the Control group, as well as for two-thirds of the electrodes in the Struc group. In the StrucDic group, all six electrodes were inserted with extended damage.

**Figure 8:**
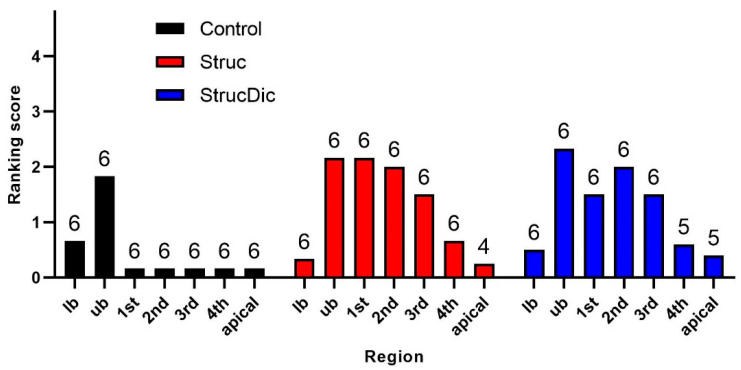
Connective tissue growth in the different turns of the cochlea. lb = lower basal; ub = upper basal turn; 1st = 1st middle turn; 2nd = 2nd middle turn; 3rd = 3rd middle turn; 4th = 4th middle turn; apical = apical turn, mean ± SD; n = small numbers on top of columns.

Newly formed connective tissue was found in all turns, from basal to apical, on the implanted side (Figure 8). In the Control group, connective tissue was mainly observed in the basal turns. In the upper basal turn, connective tissue was found in all six cochleae, with a score ranging from 1 to 3. The other turns showed a maximum score of up to 1 (less than 25% filled). The connective tissue in the Control group was loose in all six cochleae and did, in no case, fill the entire turn.

In the two treatment groups, Struc and StrucDic, relevant tissue growth was found from the upper basal turn up to the 3rd middle turn. In the Struc group, the amount of tissue varied strongly. In three of the six cochleae, most of the turns were filled with compact, partly ossified connective tissue up to a score of 4. In contrast, the other three cochleae of this group showed very little connective tissue growth in the entire cochlea (maximum score of 1).

In the StrucDic group, the distribution of the connective tissue between the cochleae was mixed. Two of the cochleae showed compact tissue (Score 4, partly ossified) in most turns. In one cochlea, more than 50% of only one turn was filled with connective tissue (Score 3). Two other cochleae were filled with connective tissue in several turns, with a score of 2. The sixth cochlea of the StrucDic group showed only little connective tissue (maximum score of 1).

Comparing the mean values of each turn between the groups, a significantly higher connective tissue score was found in the treatment groups compared to that in the Control group (Struc *p* = 0.0469; StrucDic *p* = 0.0312, according to the Wilcoxon test). No significant difference was found between the treatment groups (*p* = 0.7578). The analysis of the single values between the groups for each turn showed a significant difference in the upper basal turn (*p* = 0.0068, according to the Kruskal–Wallis test). Subsequent multiple comparisons showed significant differences in the upper basal turn between the Control and Struc (*p* = 0.0045) and the Struc and StrucDic (*p* = 0.0197) groups.

**Figure 9:**
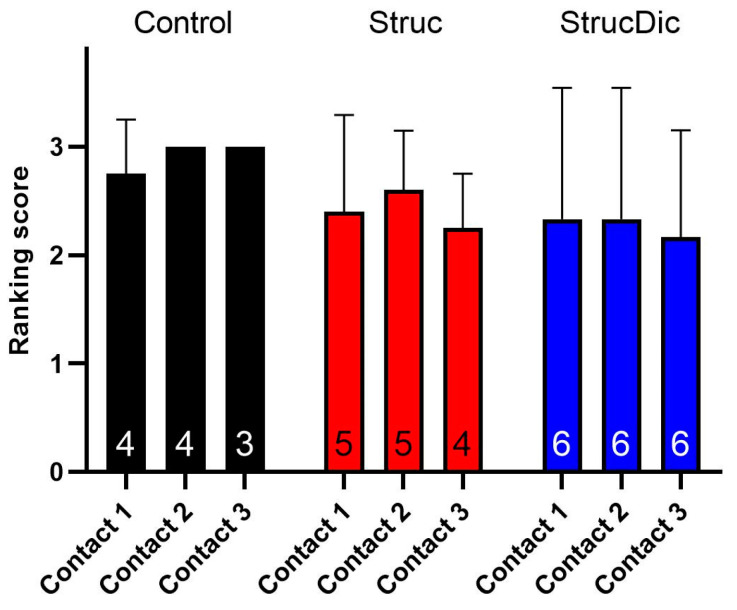
Connective tissue growth directly around the electrode contacts. Mean ± SD; n = small numbers inside columns. Contact 4 was not included in the contact score analysis due to frequent positioning in the vicinity of the cochleostomy and poor visualization.

The tissue growth at the contacts varied from none to complete occlusion with compact connective tissue. In most cases, a score of 3 was found at the contacts. In the treatment groups, a slightly higher contact score was observed at contacts 1 and 2 (Figure 9), being more deeply inserted into the cochlea than contacts 3 and 4. However, there were no significant differences between the groups. As two electrodes in the Control group slipped out during the histology evaluation, the correct allocation of connective tissue could not be fully guaranteed.

### 3.5. Spiral Ganglion Neuron Density

All presented values of SGN density were determined using the threshold setting of 0.1 for counting the SGNs with ImageJ. An overview of the mean values of SGN density for the entire cochlea are presented in Table 1. Significant differences were found between the right and the left sides in all three groups for the entire cochlea.

**Figure 10:**
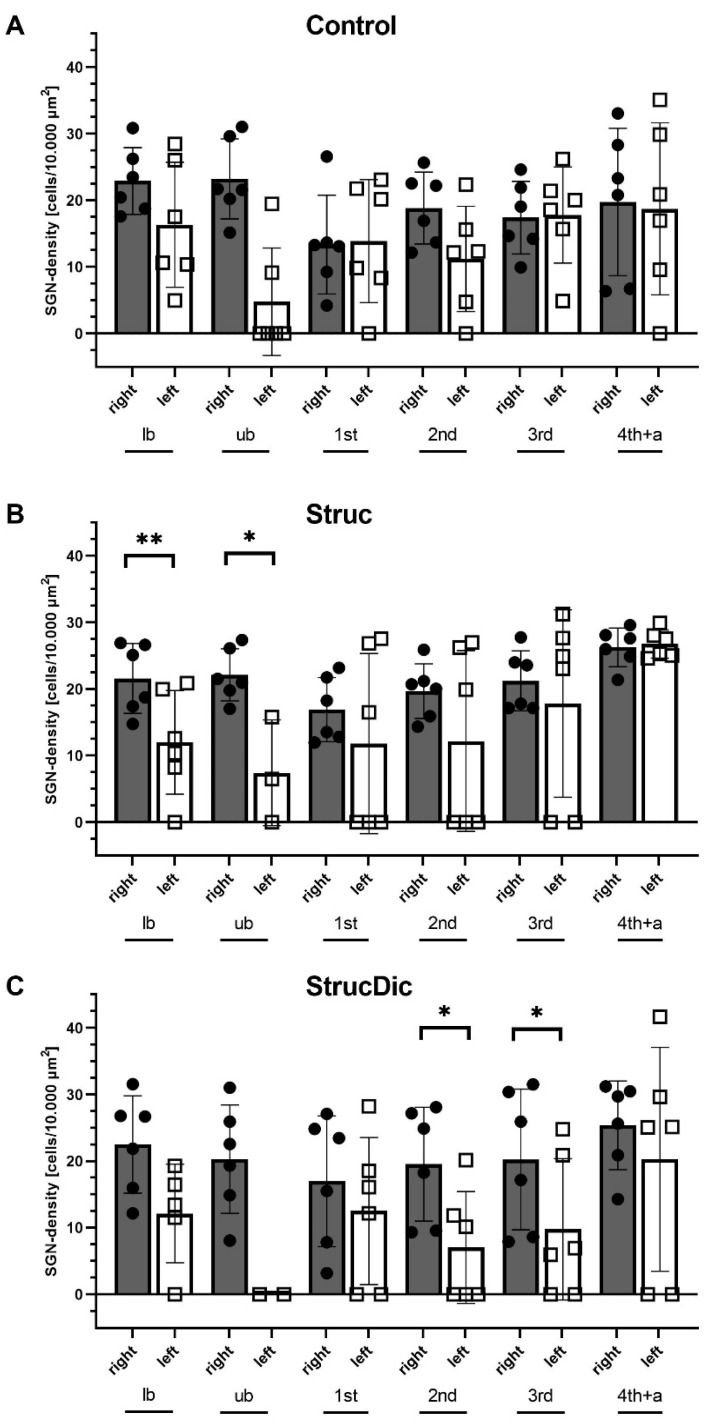
SGN density for the implanted and unimplanted side: (**A**) Control group, (**B**) Struc group, and (**C**) StrucDic group. lb = lower basal; ub = upper basal; 1st = 1st middle turn; 2nd = 2nd middle turn; 3rd = 3rd middle turn; 4th + a = 4th middle turn + apical; mean ± SD. * = *p* < 0.05; ** = *p* < 0.01.

Except for one animal at the lower basal turn in the Struc group, SGNs were found in every turn and every cochlea on the control side. In contrast, on the implanted side, sometimes, no SGNs were detected. SGNs were found in 29 out of 36 turns (Control), in 22 out of 36 turns (Struc), and in 19 out of 36 turns (StrucDic). An overview is provided in Table 2. SGNs were least abundant in the upper basal turn. Here, vital SGNs were found only in the Control and Struc group in two out of six cochleae each. In the upper basal turn of the StrucDic group, no SGNs were detected at all. Comparing the SGN density of the turns on the implanted side and the unimplanted side, no significant differences were found in the Control group (Figure 10A). In the Struc group on the implanted side, a significantly lower SGN density was observed in the basal turns (lb: *p* = 0.0086; ub: *p* = 0.0336, according to the paired *t*-tests, Figure 10B). In the StrucDic group, a significantly lower SGN density was registered in the 2nd middle turn (*p* = 0.0304, paired *t*-test, Figure 10C) and the 3rd middle turn (*p* = 0.0105, paired *t*-test) on the implanted side. As the SGN density for the upper basal turn in the StrucDic group showed values of zero for all animals, a statistical analysis could not be performed, despite the significant difference between the implanted and the unimplanted site. The SGN density on the implanted side did not show significant differences when comparing the turns between the groups.

### 3.6. Correlation Between SGN Density and Connective Tissue Score

**Figure 11:**
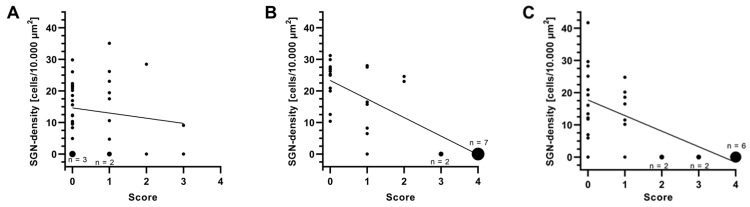
Correlation between SGN density and connective tissue score. (**A**) Control, (**B**) Struc, and (**C**) StrucDic; n = 1 (except when labelled otherwise), with the size of data points depending on n.

To evaluate a possible correlation between SGN density (Figure 10) and the connective tissue score (Figure 8), linear regression was performed. The data for SGN density were correlated with the connective tissue score for each cochlea and each turn. No significant correlation was found in the Control group (*p* = 0.4165, Figure 11A). In the Struc group, a strong negative correlation was found (r^2^ = 0.6445; *p* < 0.0001, Figure 11B). A significant negative correlation was also observed in the StrucDic group (r^2^ = 0.4451; *p* < 0.0001, Figure 11C). By correlating the SGN density of all animals of all three groups with the connective tissue score, a significant correlation was found (r^2^ = 0.3432; *p* <0.0001).

Comparing the data of the turns from all groups, significant negative correlations between SGN density and connective tissue were found in the 1st middle turn (r^2^ = 0.2442; *p* = 0.0371), in the 2nd middle turn (r^2^ = 0.5433; *p* = 0.0005), and in the 3rd middle turn (r^2^ = 0.4065; *p* = 0.0044). The other turns showed no significant correlations.

By breaking down the data of the turns into the individual groups, strong negative correlations were detected, especially in the Struc group, for the 1st middle turn (r^2^ = 0.9869; *p* < 0.0001), followed by the 2nd middle turn (r^2^ = 0.9671; *p* = 0.0004), and the 3rd middle turn (r^2^ = 0.8506; *p* = 0.0088). In the StrucDic group the SGN density and the connective tissue score only correlated significantly negatively in the 1st middle turn (r^2^ = 0.7431; *p* = 0.0272). No significant correlation was found in the turns of the Control group.

### 3.7. Correlation Between Impedances and Contact Score

**Figure 12:**
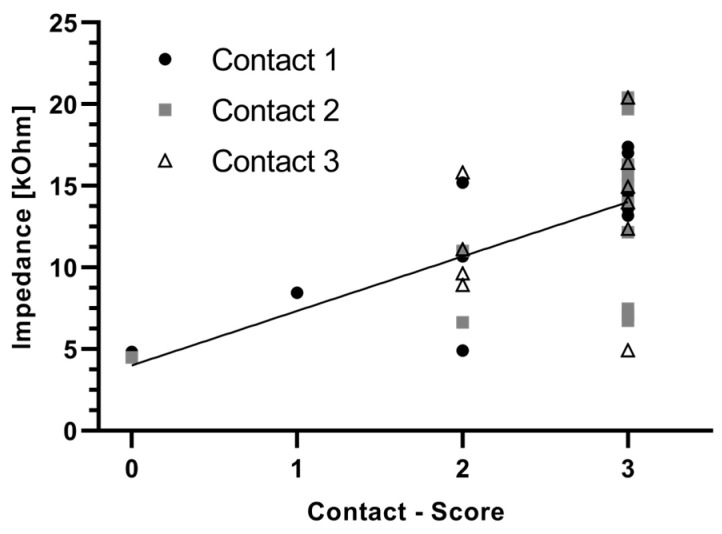
Correlation of impedances on day 28 and the respective contact score. Included are all contacts of all groups.

Linear regression was used to analyze the possible effects of the connective tissue on the electrode contact impedances. Impedance data for the contacts 1, 2, and 3 from day 28 (Figure 6) were correlated with the corresponding connective tissue score of the contacts (Figure 9). The highest positive correlation between the impedances and the connective tissue score of the contacts was found at contact 1 (r^2^ = 0.3420; *p* = 0.0220). The data from contact 2 also indicate a significant correlation (r^2^ = 0.3186; *p* = 0.0284). No significant correlation was found at contact 3 (*p* = 0.2778). When correlating the connective tissue score of all three contacts with the impedance, a significant correlation of r^2^ = 0.2782 (*p* = 0.0004, Figure 12) was also found. Within the groups, no significant correlation was found in the Control group. However, in the Struc group, the connective tissue score on the electrode contact correlated significantly with impedance at contact 1 (r^2^ = 0.7916; *p* = 0.0176), whereas in the StrucDic group, a significant correlation was found at contact 2 (r^2^ = 0.7805; *p* = 0.0196).

## 4. Discussion

The inhibition of post-implantation connective tissue growth has been the goal in many studies, as well as in the current study. To our knowledge, the effects of microstructured CI electrodes on fibroblast growth and hearing preservation have not yet been investigated.

In the current study, the silicone of all electrode arrays was loaded with 5% DEX, and microstructures were added in the treatment groups. Sustaining a long-term anti-inflammatory local dual-drug release, electrode arrays in the StrucDic group were additionally coated with PLLA loaded with diclofenac. Diclofenac was chosen because it exhibits anti-inflammatory effects and was already tested in vitro on SGNs [15,17].

In the current study, the insertion trauma approach, along with a cochleostomy, were used for increased insertional trauma, which is associated with increased connective tissue growth [4,7,35]. With both approaches, a better analysis of connective tissue inhibition was expected. Using this traumatic approach, the results showed that in ten out of twelve cases, the insertion of the electrodes in the treatment groups resulted in extended damage (either translocation of the electrode array to the scala vestibuli or even damage to the 2nd turn). However, in the Control group, extended damage was only found in one cochlea. This suggests that the surface structures of the treatment groups, with and without the additional coating, may have influenced the electrode’s mechanical properties and therefore, led to a more traumatic insertion process.

Analyzing the forces during insertion of an electrode in a three-dimensional force measurement system, Avci et al. demonstrated that insertion forces depend on friction, the angle of insertion, and the anatomy of the cochlea [36]. Additionally, a relationship between the insertion forces and the insertion speeds was reported as well [37]. As all implantations in the current study were performed by the same surgeon, the mean values for angles, forces, and speeds should be comparable between groups. Significant differences in the anatomy of the cochlea between the groups are also unlikely, as all animals came from the same breed. This leaves only the different forces caused by friction to explain the different degrees of trauma.

The microgrooves on the electrode surface have probably influenced the friction and therefore, the insertion forces. To our knowledge, there are no studies measuring the insertion forces of microstructured CI electrodes. However, there is a study calculating the friction coefficients of microgrooved polymers. Here, a glass ball that moves across a microstructured polymer surface determined the frictional behavior. The authors showed that microstructured grooves on polymers reduce friction compared to that of smooth surfaces [38]. This study is comparable to our study because the analysis of the friction coefficient was carried out perpendicular to the orientation of the grooves. These are the same conditions produced by inserting a circularly microstructured electrode into the cochlea. Furthermore, another study showed that microstructured surfaces reduce the contact angle and affect the surface roughness [39]. When comparing different microgrooves on polymers, Baum et al. showed that the friction coefficient depends on the size of the grooves. Here, the lowest friction coefficient was found using grooves 25 µm apart [38]. In the current study, the grooves were located 38 µm apart. Therefore, reduced friction using microstructured electrode arrays instead of arrays with a smooth surface could be expected.

When transferring these published results to our study, one must consider that Baum et al. and Tomanik et al. [38,39] used femtosecond laser ablation to create the microstructures, whereas molds were used in the current study. It should also be noted that both other studies comprised in vitro experiments using inorganic materials with a hard, smooth surface. In vivo, however, the inner surface of the scala tympani is not this smooth and is probably more sensitive to the microstructures. Therefore, microstructured electrodes with circular grooves might cause more damage, e.g., in the form of micro traumata during insertion, than electrodes with smooth surfaces. Here, every elevated part of the structure might have the potential to induce new trauma, which would not be the case with smooth surfaces. At least this would explain the more significant trauma found in the Struc group compared to that in the Control group.

In the StrucDic group, the electrodes were additionally coated with PLLA. Coated electrodes may have smoother surface structures than uncoated microstructured electrodes. Electrodes in the StrucDic group should therefore cause less damage to the sensitive tissue of the scala tympani. However, the results showed the opposite: When four out of six cochleae in the Struc group showed extended damage, this was the case in all six animals in the StrucDic group. This suggests that the PLLA coating may affect the mechanical properties of the electrode, resulting in higher insertion forces and more damage to the cochlea.

A recent study showed that PLLA-coated electrodes do not increase insertion forces when inserted into a linear in vitro model of the scala tympani [26]. However, the increased damage with a PLLA-coated electrode reported in our study is consistent with the results of an in vivo study using PLLA-coated electrodes [18]. This suggests that while the PLLA coating may not increase the insertion forces in a linear model, the stiffened electrode may still lead to increased damage in a cochlea with several turns [26].

Our results from the histological evaluation showed that the formation of new connective tissue ranged from almost none, adjacent to the electrode, to almost all turns of a cochlea completely filled with compact connective tissue. This extensive connective tissue growth appears to be linked to a whole-cochlear inflammatory response, which was absent in the Control group but present in most cases in the treatment groups.

As described above, the electrodes in the treatment groups caused more damage within the cochlea; in some cases, the scala vestibuli or even the second turn were affected. During insertion of the CI electrode, there may have been increased transport of bone fragments, dust (from cochleostomy drilling), and/or blood into the upper parts of the cochlea, triggering a foreign body reaction, especially as a trauma model with multiple insertions was used in the current study [40,41,42]. This could explain the whole-cochlear response that occurred in some animals. The immune response to a foreign body is characterized by the accumulation of lymphocytes and macrophages [43]. In some cases, macrophages begin to fuse and form foreign-body giant cells (FBGCs), assisting the macrophages in phagocytosing foreign material [44]. Since FBGCs work like osteoblasts, this would explain the osseous tissue found in some cochleae [45]. This is consistent with the work of O’Leary et al., who reported increased scala tympani occlusion and the presence of FBGCs when CI electrodes were inserted through a cochleostomy [5].

Previous in vitro studies showed that microstructured surfaces can affect cells in various ways, such as by influencing cell morphology, promoting nerve cell guidance, or promoting and inhibiting cell attachment [28,29,46]. In the current study, we investigated a possible reduction in connective tissue growth using microstructured electrode arrays. Despite the generated damage, the results showed that some of the cochleae exhibited very little connective tissue, even after the implantation of microstructured electrodes. However, due to the severe trauma induced by the traumatic insertion approach chosen, a clear judgement of the effect of microstructured CI surfaces on connective tissue formation cannot be provided.

Average hearing thresholds increased by more than 50 dB on the implanted side compared to the unimplanted side (6 dB) at day 28. Since a relationship between connective tissue and increased hearing threshold has previously been described in the literature, the results may be explained by the presence of connective tissue on the implanted side but also by the generated damage [5]. Our results are consistent with those of Behrends et al., who reported threshold shifts of up to 60 dB, but not with the results of Ceschi et al., who reported lower absolute threshold shifts of up to 40 dB [18,25]. In both studies, PLLA-coated electrodes were used in vivo. However, Ceschi et al. performed an atraumatic insertion of electrodes without contact and wires through the round window membrane. The two other studies used a traumatic insertion approach combined with a cochleostomy. The combination of differently shaped electrodes [9], a more traumatic insertion [4], and noise damage due to drilling the cochleostomy [47] resulted in higher threshold shifts and may explain the discrepancy with the results reported by Ceschi et al. [25]. The higher hearing thresholds can therefore be explained by the increased damage and are not necessarily associated with the surface structures, coatings, or drugs applied. However, we must acknowledge that we still do not know enough about the exact mechanisms leading to threshold shifts.

The impedances in the three groups increased to values between 11 and 12.5 kOhm on day 28. Especially in the Control group, the absolute values were very similar to those reported by Wilk et al., who used electrodes with different concentrations of DEX incorporated into the silicone array in vivo [4]. Another in vivo study using diclofenac-loaded PLLA-coated electrodes showed impedances very similar to those in the current study [18]. Since both studies used the trauma approach, the results are comparable.

In the current study, there were no significant differences in impedances between the groups, despite the differences in the connective tissue. As the electrodes were positioned in the upper basal turn, where the differences in connective tissue between the groups were small, this may explain the minor differences in impedance between the groups.

The connective tissue score directly around the first three contacts (contact score) was also very similar between the groups. Although the electrodes each had four contacts, contact 4 was not included in the contact score analysis due to frequent positioning in the vicinity of the cochleostomy and poor visualization. Additionally, some of the electrodes in the Control group (two electrodes) and in the Struc group (one electrode) slipped out during the histological preparation. Remarkably, cochleae with electrode dislocation during sample preparation showed very little connective tissue, suggesting that the electrode was less likely to be anchored in these cases.

As impedances are influenced by the surrounding connective tissue [4], in the current study, a correlation between impedance and contact score was evaluated. Significant correlations between impedance and connective tissue directly around the electrode contacts were found for contact 1, contact 2, and for all three contacts together. This suggests that impedances significantly correlate with the amount of connective tissue directly around the electrode contacts.

The mean SGN density on the non-implanted side was approximately 20 SGNs/10,000 µm^2^, which is consistent with the results of previous publications using both confocal laser scanning microscopy and the ImageJ counting plug-in ITCN [18,34].

However, the ITCN plug-ins available on the Internet vary in their ability to set a threshold. Both plug-ins allow for the automatic counting of the number of SGNs, but only in the plug-in provided by the Center for Bio-image Informatics (UC Santa Barbara) we found the possibility to set a threshold of 0.1. Without this setting, the threshold is preset to 0.0. In the current study, the different thresholds were compared with a manual count. The results showed that the 0.1 threshold is comparable to the manual count, whereas the 0.0 threshold showed significantly higher counts. For accurate results, therefore, the correct plug-in, or a correction factor of 0.63 for the whole cochlea to convert the values from the 0.0 setting to the 0.1 setting, should be used to compare the results from different studies.

The results showed significant differences in SGN density between the treated and the untreated side in all three groups for the entire cochlea. Additionally, in the basal turns of the Struc and StrucDic groups, and for the higher turns of the StrucDic group, significant differences were found when comparing the turns of the implanted side with those of the untreated side. It may be speculated that the reduction in SGNs may be caused by the increased trauma and the strong inflammatory reaction triggering the development of compact connective tissue. The significant negative correlations between SGN density and connective tissue found for all animals and all turns (*p* = 0.0002; r^2^ = 0.1219), and especially in the Struc group, suggest that connective tissue growth might have a negative impact on SGN survival in vivo.

## 5. Conclusions

The microstructured electrodes employed appear to have contributed to increased damage within the cochlea due to the individual elevated parts of the structure. However, despite the microstructures, there are also animals in both treatment groups with very little connective tissue. This suggests that the increased damage due to the trauma model in the current study may have superimposed a possible inhibition of connective tissue formation. This is supported by the connective tissue results, which showed significant differences between the Control and treatment groups.

The analysis of the impedances and hearing thresholds showed no long-term reduction between the groups. The SGN survival analysis also showed no significant protection obtained by the applied microstructures and the dual-drug release. However, there was a significant correlation between the impedances and the amount of connective tissue around the electrode contacts. In addition, negative correlations were found between connective tissue and SGN survival. However, to reduce the superimposition of possible inhibitory effects, we would recommend using microstructured electrode arrays, without the trauma model.

## Figures and Tables

**Table 1:** Values of SGN density for the entire cochlea in cells per 10,000 µm^2^. Mean ± SD.

Group	Unimplanted Side	Implanted Side	*p*-Value
Control	19.2 ± 7.4	13.8 ± 10	0.0231
Struc	21.3 ± 4.9	15.3 ± 11.8	0.0084
StrucDic	20.8 ± 8.4	11.5 ± 12	0.0002

**Table 2:** Total number of turns in which SGNs were detected on the implanted side.

SGNs on Implanted Side	Control	Struc	StrucDic	Sum
Lower basal turn	6/6	5/6	4/6	15/18
Upper basal turn	2/6	2/6	0/6	4/18
1st middle turn	5/6	3/6	4/6	12/18
2nd middle turn	5/6	3/6	3/6	11/18
3rd middle turn	6/6	3/6	4/6	13/18
4th middle turn + apical	5/6	6/6	4/6	15/18

## Data Availability

The original contributions presented in this study are included in the article. Further inquiries can be directed to the corresponding author.

## References

[1] Swiderski D.L., Colesa D.J., Hughes A.P., Raphael Y., Pfingst B.E. (2020). Relationships between Intrascalar Tissue, Neuron Survival, and Cochlear Implant Function. JARO.

[2] Kamakura T., O’Malley J.T., Nadol J.B. (2018). Preservation of Cells of the Organ of Corti and Innervating Dendritic Processes Following Cochlear Implantation in the Human: An Immunohistochemical Study. Otol. Neurotol..

[3] Xu J., Shepherd R.K., Millard R.E., Clark G.M. (1997). Chronic Electrical Stimulation of the Auditory Nerve at High Stimulus Rates: A Physiological and Histopathological Study. Hear. Res..

[4] Wilk M., Hessler R., Mugridge K., Jolly C., Fehr M., Lenarz T., Scheper V. (2016). Impedance Changes and Fibrous Tissue Growth after Cochlear Implantation Are Correlated and Can Be Reduced Using a Dexamethasone Eluting Electrode. PLoS ONE.

[5] O’Leary S.J., Monksfield P., Kel G., Connolly T., Souter M.A., Chang A., Marovic P., O’Leary J.S., Richardson R., Eastwood H. (2013). Relations between Cochlear Histopathology and Hearing Loss in Experimental Cochlear Implantation. Hear. Res..

[6] Somdas M.A., Li P.M.M.C., Whiten D.M., Eddington D.K., Nadol J.B. (2007). Quantitative Evaluation of New Bone and Fibrous Tissue in the Cochlea Following Cochlear Implantation in the Human. Audiol. Neurotol..

[7] Fayad J.N., Makarem A.O., Linthicum F.H. (2009). Histopathologic Assessment of Fibrosis and New Bone Formation in Implanted Human Temporal Bones Using 3D Reconstruction. Otolaryngol.—Head Neck Surg..

[8] Hrnčiřík F., Nagy L., Grimes H.L., Iftikhar H., Muzaffar J., Bance M. (2024). Impact of Insertion Speed, Depth, and Robotic Assistance on Cochlear Implant Insertion Forces and Intracochlear Pressure: A Scoping Review. Sensors.

[9] Adunka O., Unkelbach M.H., MacK M., Hambek M., Gstoettner W., Kiefer J. (2004). Cochlear Implantation via the Round Window Membrane Minimizes Trauma to Cochlear Structures: A Histologically Controlled Insertion Study. Acta Oto-Laryngol..

[10] Rebscher S.J., Hetherington A., Bonham B., Wardrop P., Leake P.A. (2008). Considerations for the Design of Future Cochlear Implant Electrode Arrays: Electrode Array Stiffness, Size and Depth of Insertion. J. Rehabil. Res. Dev..

[11] Farhadi M., Jalessi M., Salehian P., Ghavi F.F., Emamjomeh H., Mirzadeh H., Imani M., Jolly C. (2013). Dexamethasone Eluting Cochlear Implant: Histological Study in Animal Model. Cochlear Implant. Int..

[12] Nieratschker M., Liepins R., Honeder C., Auinger A.B., Gausterer J.C., Baumgartner W.-D., Riss D., Arnoldner C., Dahm V. (2024). A Single Intratympanic Triamcinolone Acetonide Administration Elicits Long-Term Reduction in Impedances Following Cochlear Implantation. J. Otolaryngol.—Head Neck Surg..

[13] Stathopoulos D., Chambers S., Enke Y.L., Timbol G., Risi F., Miller C., Cowan R., Newbold C. (2014). Development of a Safe Dexamethasone-Eluting Electrode Array for Cochlear Implantation. Cochlear Implant. Int..

[14] Briggs R., O’Leary S., Birman C., Plant K., English R., Dawson P., Risi F., Gavrilis J., Needham K., Cowan R. (2020). Comparison of Electrode Impedance Measures between a Dexamethasone-Eluting and Standard Cochlear^TM^ Contour Advance^®^ Electrode in Adult Cochlear Implant Recipients. Hear. Res..

[15] Al-Nimer M.S.M., Hameed H.G., Mahmood M.M. (2015). Antiproliferative Effects of Aspirin and Diclofenac against the Growth of Cancer and Fibroblast Cells: In Vitro Comparative Study. Saudi Pharm. J..

[16] Srivastava R., Jayant R.D., Chaudhary A., McShane M.J. (2011). “Smart Tattoo” Glucose Biosensors and Effect of Coencapsulated Anti-Inflammatory Agents. J. Diabetes Sci. Technol..

[17] Wulf K., Goblet M., Raggl S., Teske M., Eickner T., Lenarz T., Grabow N., Paasche G. (2022). PLLA Coating of Active Implants for Dual Drug Release. Molecules.

[18] Behrends W., Wulf K., Raggl S., Fröhlich M., Eickner T., Dohr D., Esser K.-H., Lenarz T., Scheper V., Paasche G. (2023). Dual Drug Delivery in Cochlear Implants: In Vivo Study of Dexamethasone Combined with Diclofenac or Immunophilin Inhibitor MM284 in Guinea Pigs. Pharmaceutics.

[19] Eshraghi A.A., Dinh C.T., Bohorquez J., Angeli S., Abi-Hachem R., Van De Water T.R. (2011). Local Drug Delivery to Conserve Hearing: Mechanisms of Action of Eluted Dexamethasone within the Cochlea. Cochlear Implant. Int..

[20] Creber N.J., Eastwood H.T., Hampson A.J., O’Leary S.J. (2023). Cochlear Implant Surgery Facilitates Intracochlear Distribution of Perioperative Systemic Steroids. Acta Oto-Laryngol..

[21] Borenstein J.T. (2011). Intracochlear Drug Delivery Systems. Expert Opin. Drug Deliv..

[22] Salt A.N., Hartsock J., Plontke S., LeBel C., Piu F. (2011). Distribution of Dexamethasone and Preservation of Inner Ear Function Following Intratympanic Delivery of a Gel-Based Formulation. Audiol. Neurotol..

[23] Huang Y., Yu H., Liang M., Hou S., Chen J., Zhang F., Sun X., Jia H., Yang J. (2021). Hearing Protection Outcomes of Analog Electrode Arrays Coated with Different Drug-Eluting Polymer Films Implanted into Guinea Pig Cochleae. DDDT.

[24] Bohl A., Rohm H.W., Ceschi P., Paasche G., Hahn A., Barcikowski S., Lenarz T., Stöver T., Pau H.-W., Schmitz K.-P. (2012). Development of a Specially Tailored Local Drug Delivery System for the Prevention of Fibrosis after Insertion of Cochlear Implants into the Inner Ear. J. Mater. Sci. Mater. Med..

[25] Ceschi P., Bohl A., Sternberg K., Neumeister A., Senz V., Schmitz K.P., Kietzmann M., Scheper V., Lenarz T., Stöver T. (2014). Biodegradable Polymeric Coatings on Cochlear Implant Surfaces and Their Influence on Spiral Ganglion Cell Survival. J. Biomed. Mater. Res..

[26] Dohr D., Wulf K., Grabow N., Mlynski R., Schraven S.P. (2022). A PLLA Coating Does Not Affect the Insertion Pressure or Frictional Behavior of a CI Electrode Array at Higher Insertion Speeds. Materials.

[27] Stefanini G.G., Byrne R.A., Serruys P.W., De Waha A., Meier B., Massberg S., Juni P., Schomig A., Windecker S., Kastrati A. (2012). Biodegradable Polymer Drug-Eluting Stents Reduce the Risk of Stent Thrombosis at 4 Years in Patients Undergoing Percutaneous Coronary Intervention: A Pooled Analysis of Individual Patient Data from the ISAR-TEST 3, ISAR-TEST 4, and LEADERS Randomized Trials. Eur. Heart J..

[28] Wójciak-Stothard B., Curtis A.S.G., McGrath M., Sommer I., Wilkinson C.D.W., Monaghan W. (1995). Role of the Cytoskeleton in the Reaction of Fibroblasts to Multiple Grooved Substrata. Cell Motil. Cytoskelet..

[29] Thakar R.G., Cheng Q., Patel S., Chu J., Nasir M., Liepmann D., Komvopoulos K., Li S. (2009). Cell-Shape Regulation of Smooth Muscle Cell Proliferation. Biophys. J..

[30] Van Kooten T.G., Whitesides J.F., Von Recum A.F. (1998). Influence of Silicone (PDMS) Surface Texture on Human Skin Fibroblast Proliferation as Determined by Cell Cycle Analysis. J. Biomed. Mater. Res..

[31] Tykocinski M., Duan Y., Tabor B., Cowan R.S. (2001). Chronic Electrical Stimulation of the Auditory Nerve Using High Surface Area (HiQ) Platinum Electrodes. Hear. Res..

[32] Maslan M.J., Miller J.M. (1987). Electrical stimulation of the guinea pig cochlea. Otolaryngol.—Head Neck Surg..

[33] MacDonald G.H., Rubel E.W. (2008). Three-Dimensional Imaging of the Intact Mouse Cochlea by Fluorescent Laser Scanning Confocal Microscopy. Hear. Res..

[34] Wrzeszcz A., Reuter G., Nolte I., Lenarz T., Scheper V. (2013). Spiral Ganglion Neuron Quantification in the Guinea Pig Cochlea Using Confocal Laser Scanning Microscopy Compared to Embedding Methods. Hear. Res..

[35] Bas E., Bohorquez J., Goncalves S., Perez E., Dinh C.T., Garnham C., Hessler R., Eshraghi A.A., Van De Water T.R. (2016). Electrode Array-Eluted Dexamethasone Protects against Electrode Insertion Trauma Induced Hearing and Hair Cell Losses, Damage to Neural Elements, Increases in Impedance and Fibrosis: A Dose Response Study. Hear. Res..

[36] Avci E., Nauwelaers T., Hamacher V., Kral A. (2017). Three-Dimensional Force Profile During Cochlear Implantation Depends on Individual Geometry and Insertion Trauma. Ear Hear..

[37] Kaufmann C.R., Henslee A.M., Claussen A., Hansen M.R. (2020). Evaluation of Insertion Forces and Cochlea Trauma Following Robotics-Assisted Cochlear Implant Electrode Array Insertion. Otol. Neurotol..

[38] Baum M.J., Heepe L., Fadeeva E., Gorb S.N. (2014). Dry Friction of Microstructured Polymer Surfaces Inspired by Snake Skin. Beilstein J. Nanotechnol..

[39] Tomanik M., Kobielarz M., Filipiak J., Szymonowicz M., Rusak A., Mroczkowska K., Antończak A., Pezowicz C. (2020). Laser Texturing as a Way of Influencing the Micromechanical and Biological Properties of the Poly(L-Lactide) Surface. Materials.

[40] Smouha E.E. (2003). Surgery of the Inner Ear With Hearing Preservation: Serial Histological Changes. Laryngoscope.

[41] Simoni E., Gentilin E., Candito M., Borile G., Romanato F., Chicca M., Nordio S., Aspidistria M., Martini A., Cazzador D. (2020). Immune Response After Cochlear Implantation. Front. Neurol..

[42] Radeloff A., Unkelbach M.H., Tillein J., Braun S., Helbig S., Gstöttner W., Adunka O.F. (2007). Impact of Intrascalar Blood on Hearing. Laryngoscope.

[43] O’Malley J.T., Burgess B.J., Galler D., Nadol J.B. (2017). Foreign Body Response to Silicone in Cochlear Implant Electrodes in the Human. Otol. Neurotol..

[44] Anderson J.M., Rodriguez A., Chang D.T. (2008). Foreign Body Reaction to Biomaterials. Semin. Immunol..

[45] Barbeck M., Booms P., Unger R., Hoffmann V., Sader R., Kirkpatrick C.J., Ghanaati S. (2017). Multinucleated Giant Cells in the Implant Bed of Bone Substitutes Are Foreign Body Giant Cells—New Insights into the Material-mediated Healing Process. J. Biomed. Mater. Res..

[46] Cai L., Zhang L., Dong J., Wang S. (2012). Photocured Biodegradable Polymer Substrates of Varying Stiffness and Microgroove Dimensions for Promoting Nerve Cell Guidance and Differentiation. Langmuir.

[47] Pau H.W., Just T., Bornitz M., Lasurashvilli N., Zahnert T. (2007). Noise Exposure of the Inner Ear During Drilling a Cochleostomy for Cochlear Implantation. Laryngoscope.

